# Treatment of Sprains

**Published:** 1901-07

**Authors:** 


### Treatment of Sprains.

Dr. Haldor Sneve contributes an exceedingly interesting article on the treatment of muscular and joint sprains. He objects to the method in common use of immobilizing the joint, on the ground that immobilization for a considerable time produces passive inflammatory changes and favors absorption of the synovial fluid, with roughening of the joint surfaces, and also atrophy and contraction of the muscles of the part together with absorption of the fat and areolar tissue around the ligaments and tendons.

His routine method of treatment in these cases is elevation of the part, the application of a wet cheese cloth dressing and an ice bag until the height of the inflammatory process is reached. Then, after the subsidence of the acute symptoms, he uses hot fomentation and massage to assist the removal of inflammatory products. As soon as possible he has the patient take active exercise. By this treatment, he believes that the time ordinarily required for recovery can be shortened at least fifty per cent.—*Journal of the American Medical Association,* June 1, 1901.

